# CircPRMT5 circular RNA promotes proliferation of colorectal cancer through sponging miR‐377 to induce E2F3 expression

**DOI:** 10.1111/jcmm.15019

**Published:** 2020-02-05

**Authors:** Bairen Yang, Ke Du, Chuanhua Yang, Lili Xiang, Ying Xu, Chen Cao, Junhui Zhang, Wenneng Liu

**Affiliations:** ^1^ Department of General Surgery The First People's Hospital of Yibin Yibin China; ^2^ BioBank The First Affiliated Hospital of Xi'an Jiaotong University Shaanxi China; ^3^ West China School of Public Health and West China Fourth Hospital Sichuan University Chengdu China

**Keywords:** cell cycle, CircPRMT5, CRC, E2F3, miR‐377

## Abstract

CircPRTM5 is associated with cell proliferation and migration in many kinds of malignancies. However, the functions and mechanisms of CircPRTM5 in CRC progression remain unclear. We explored the role and the mechanisms of CircPRTM5 in the development of CRC. Tissues of CRC patients and matched adjacent non‐tumour tissues were collected to evaluate the expression of CircPRTM5. The expression of CircPRTM5 in CRC tissues was significantly higher than that in adjacent tissues. The biological functions of CircPRTM5 in CRC were determined by overexpression and down‐regulation of CircPRTM5 in CRC cells in vitro and in vivo. The results indicate that knockdown of CircPRTM5 can significantly inhibit the proliferation of CRC cells. The potential mechanisms of CircPRTM5 in CRC development were identified by RT‐qPCR, Western blotting analysis and luciferase reporter assay. CircPRTM5 competitively regulates the expression of E2F3 by capillary adsorption of miR‐377. CircPRMT5 regulates CRC proliferation by regulating the expression of E2F3, which affects the expression of the cell cycle‐associated proteins cyclinD1 and CDK2. CircPRTM5 exerts critical regulatory role in CRC progression by sponging miR‐377 to induce E2F3 expression.

## 1 INTRODUCTION

Colorectal cancer (CRC) ranks one of the most common malignancies worldwide. The mortality of CRC ranks the third highest in all types of cancer, bringing a great burden to humans.^1^, ^2^, ^3^ However, the molecular basis of CRC still largely unknown.

Circular RNAs (circRNAs) have been known as a class of widespread and diverse endogenous small RNAs with multiple regulatory roles. CircRNAs are possessed with covalently closed loop structure that are formed from Exon ‘skipping’ and ‘direct back‐splicing’ of pre‐mRNA transcripts.^4^, ^5^ It is well documented that circRNAs are usually exerts function as miRNA sponges to trap miRNA in the process of tumorigenesis.^6^ Evidence suggested that circHIPK3 could involve in cell proliferation by sponging multiple miRNAs.^7^ Recent study confirmed that circSMARCA5/miR‐17‐3p/miR‐181b‐5p axis suppresses the proliferation and migration of hepatocellular carcinoma (HCC), making circSMARCA5 a potential therapeutic target of HCC.^8^ However, the potential functions and mechanisms of dysregulated circRNAs involved in the progression of CRC remain unclear.

In this study, we identified that circPRTM5 was frequently up‐regulated in CRC tissues and patients with higher circPRTM5 levels showed a poorer overall survival. CircPRTM5 exerts critical role in CRC by sponging miR‐377 to induce E2F3 expression and promoting cyclinD1 and CDK2 expression, which opens up a new insight into the potential treatment methods of CRC in humans.

## 2 MATERIALS AND METHODS

### 2.1 Human samples

Colorectal cancer samples and matched adjacent non‐tumour samples were gained from surgery patients of the Fourth Hospital of Western China. H&E stained slides were analysed in blind by pathologists. All patients were signed with written informed consent according to the Clinical Research Ethics Committee of the Fourth Hospital of Western China.

### 2.2 Animal models of colorectal cancer

All procedures of animal models were approved by the Institutional Animal Care and Use of Fourth Hospital of Western China. Each group of nude mice (male, 6‐week‐old, BALB/C strain) was injected subcutaneously with 5 × 10^6^ cells in 0.2 mL PBS. Tumour volume and tumour weight were measured. Tumours were collected and prepared for histological examination.^9^

### 2.3 Cell lines and cell culture

Human CRC cell lines HCT‐116, LoVo, SW‐480, SW‐620, HT‐29 and immortalized colon epithelial cell line FHC were purchased from Cell bank of Chinese Academy of Sciences. All the cells were cultured in a DMEM medium (Gibco, USA) supplemented with 10% foetal bovine serum (Gibco) and 100 U/mL penicillin/streptomycin at 37°C in a humidified incubator containing 5% CO^2^.^10^

### 2.4 Statistical analysis

Statistical assay was performed by using SPSS 20.0 software (SPSS). All the experiment was performed in triplicate. Student's *t* test was used to explain differences between two groups, and all the quantitative data were indicated as mean ± SD. For a single comparison, two‐sided *P* < .05 was considered as statistically significant.

## 3 RESULTS

### 3.1 CircPRMT5 was up‐regulated in CRC

**Figure 1:**
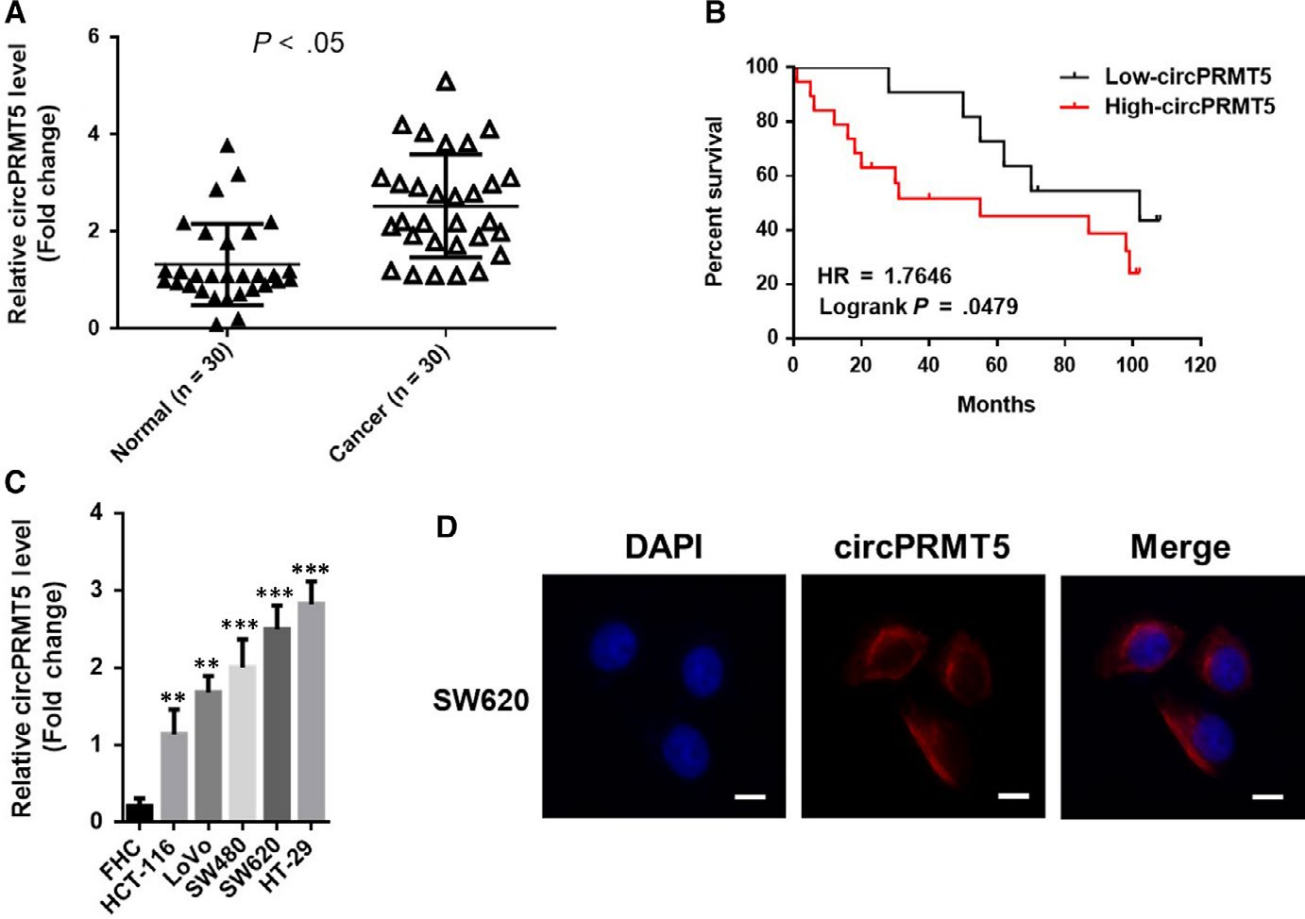
CircPRMT5 was up‐regulated in CRC and correlates with poor prognosis. A, CircPRMT5 levels were increased in CRC tissues compared with paired adjacent non‐tumour tissues as determined by RT‐qPCR. Data are mean ± SD. n = 30; B, Kaplan‐Meier curve depicting the overall survival of CRC patients (n = 30). The curves were stratified based on the CircPRMT5 level. Overall survival was defined as the interval between the date of surgery and the date of death or last follow‐up; C, CircPRMT5 levels were increased in CRC cells compared with immortalized human colon epithelial cell line FHC as determined by RT‐qPCR. Data are mean ± SD. **P < .01, ***P < .001; D, representative FISH images of circPRMT5, showing localization of circPRMT5 in the cytoplasm of SW620 cells. Scale bars, 10 μm

To investigate the expression level and location of CircPRMT5 in CRC tissues, we analysed CRC tissue samples and their matched non‐tumour tissue samples (n = 30) by using RT‐qPCR, and validated that the level of circPRMT5 was frequently elevated in CRC tissues compared with paired non‐tumour tissues (Figure 1A). Moreover, patients with higher circPRTM5 levels showed a poorer overall survival (Figure 1B), suggesting that circPRTM5 may involve in the pathological process of CRC. Analysing the clinical data of CRC patients, we found that the expression of CircPRMT5 was correlated with the patient's age, tumour size, depth of invasion and AJCC stage (Table 1). We further characterized the expression and distribution of circPRTM5 in CRC cells. Compared with FHC cell line (an immortalized human colon epithelial cell line), the expression level of circPRMT5 was significantly up‐regulated in five CRC cell lines (Figure 1C). Further examination by FISH revealed that the circPRMT5 primary expressed in the cytoplasm of CRC cells (Figure 1D). Therefore, our data showed that circPRMT5 is significantly overexpressed in human CRC tissues, as well as CRC cell lines.

**Table 1:** CircPRMT5 levels and clinicopathological features in 30 CRC patients

Characteristics	Total	CircPRMT5		*P*
		Low	High	
Gender				
Female	14	7	7	1.000
Male	16	7	9	
Age (y)				
＞60	17	4	13	.010
≤60	13	3	10	
Tumour size(cm)				
＜5	13	11	2	.001
≥5	17	4	13	
Invasion depth				
Without Infiltration into Serous layer	20	9	11	.010
Infiltration into Serous layer	10	1	9	
AJCC stage				
I\II	12	8	4	.001
III\IV	18	2	16	

### 3.2 CircPRMT5 promoted CRC cells proliferation

**Figure 2:**
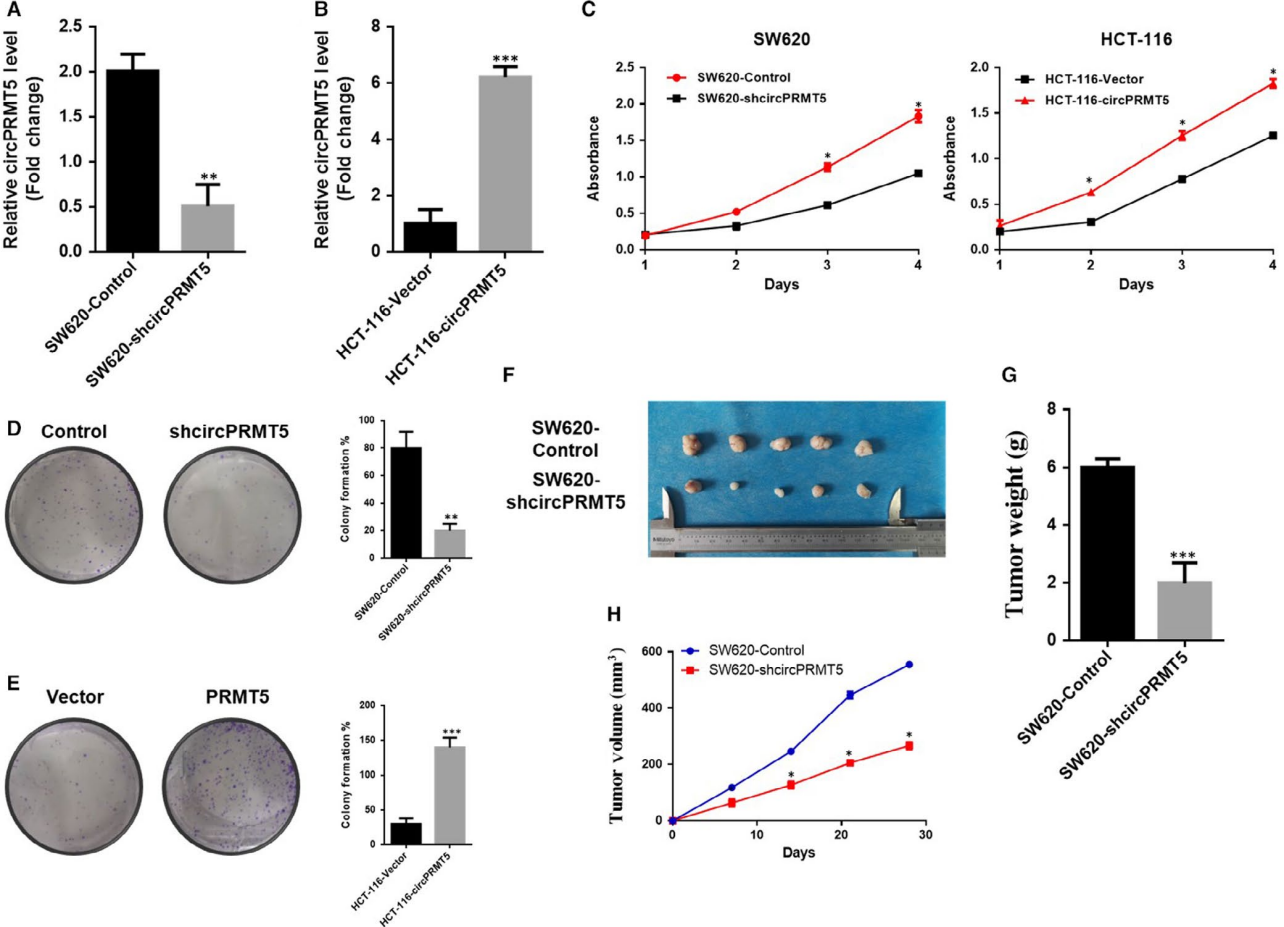
CircPRMT5 promoted cell proliferation of CRC in vitro and in vivo. (A). RT‐qPCR for circPRMT5 in SW620 cells was transfected with the shRNAs. Data are mean ± SD. **P < .01; (B). RT‐qPCR for circPRMT5 in HCT‐116 cells transfected with control vector or circPRMT5 overexpression plasmid. Data are mean ± SD. ***P < .001; (C). CCK‐8 assay of CRC cells overexpression of circPRMT5 or down‐regulation of circPRMT5. Data are mean ± SD. *P < .05; (D and E). colony formation assay of CRC cells overexpression of circPRMT5 or down‐regulation of circPRMT5. Data are mean ± SD. **P < .01, ***P < .001; (F). representative images of tumours injected with SW620‐control cells or SW620‐shcircPRMT5 cells; (G). tumour weight in the xenograft model. Data are mean ± SD. n = 5/group. ***P < .001; (H). Growth curve of tumour volumes in the xenograft model. Data are mean ± SD. n = 5/group. *P < .05

We thus evaluated the biological functions of circPRMT5 in CRC by generating SW‐620‐shcircPRMT5 cells (Figure 2A) and HCT‐116‐circPRMT5 cells (Figure 2B). The results showed that depletion of circPRMT5 substantially decreased cell proliferation capacity in CRC cells, while overexpression of circPRMT5 significantly promoted cell proliferation capacity in CRC cells as determined by CCK‐8 assay (Figure 2C). What is more, depletion of circPRMT5 showed a decrease of colonies (Figure 2D), while overexpression of circPRMT5 promoted the colony formation in CRC cells (Figure 2E). Coherent with the results in vitro, subcutaneous xenograft nude mouse models also validated that circPRMT5 significantly promoted tumorigenicity of CRC as evidenced by increase in tumour size (Figure 2F), tumour weight (Figure 2G) and tumour volume (Figure 2H). These results indicated that circPRMT5 is important in cell proliferation of CRC.

### 3.3 CircPRMT5 promoted CRC proliferation by regulating cell cycle‐associated proteins

**Figure 3:**
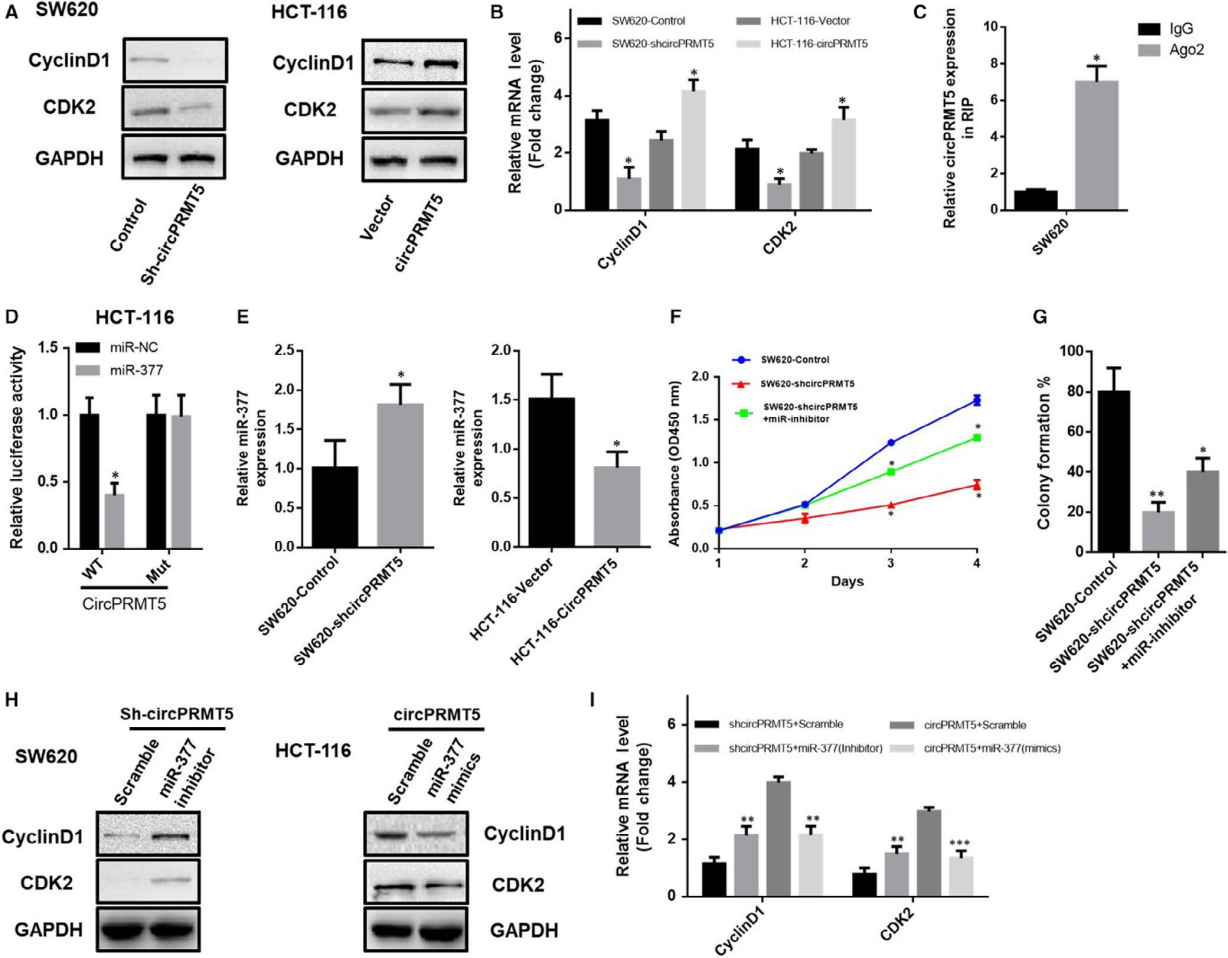
CircPRMT5 promoted CRC by reducing the inhibitory effect of miR‐377 by sponge activity. (A). Protein expression of CDK2 and cyclinD1 was determined by Western blotting; (B). mRNA expression of CDK2 and cyclinD1 was determined by RT‐qPCR. Data are mean ± SD. *P < .05; (C). Ago2 RNA immunoprecipitation (RIP) assay for the amount of circPRM5 in CRC cells expressing Flag‐AGO2 or Flag‐GFP. Data are mean ± SD. *P < .05; (D). Luciferase reporter assay for the luciferase activity of LUC‐circPRMT5 or LUC‐circPRMT5‐mutant in HCT‐116 cells cotransfected with miR‐377 mimics. Data are mean ± SD. *P < .05; (E). RNA pull down assay for the amount of circPRMT5 and miR‐377 with circPRMT5 probe in SW620 cells expressing either control or circPRMT5 shRNA or in HCT‐116 cells expressing either with control vector or circPRMT5 overexpression plasmid. Data are mean ± SD. *P < .05; (F). CCK‐8 assay of SW620 cells transfected with the shcircPRMT5 within or without cotransfected with miR‐377 inhibitor. Data are mean ± SD. *P < .05; (G). Colony formation assay of SW620 cells transfected with the shcircPRMT5 within or without cotransfected with miR‐377 inhibitor. Data are mean ± SD. *P < .05, **P < .01; (H). Western blotting analysis of CyclinD1 and CDK2 in SW620 cells transfected with the shcircPRMT5 or HCT‐116 cells expressing with circPRMT5 overexpression plasmid; (I). RT‐qPCR analysis of CyclinD1 and CDK2 in CRC cells transfected with the shcircPRMT5 or with circPRMT5 overexpression plasmid, and within or without cotransfected with miR‐377 mimics or inhibitor. Data are mean ± SD. **P < .01, ***P < .001

To deeply investigate the mechanism of circPRMT5 on cell growth in CRC, we examined the cell cycle‐associated proteins known to up‐regulated in CRC progression. RT‐PCR and Western bolt results showed that there was increase in *Cyclin*D1 and CDK2 expression when the cells overexpressed circPRMT5 (Figure 3A,B), suggesting that cell cycle‐associated proteins *Cyclin*D1 and CDK2 are involved in the oncogenic process of circPRMT5 in CRC.

### 3.4 CircPRMT5 promoted CRC by reducing the inhibitory effect of miR‐377 by sponge activity

Evidence suggested that circPRMT5 usually act as miRNA sponge to exert its function in tumorigenesis.^6^ MiR‐377, miR‐30c, miR‐188, miR‐335 and miR‐597 were reported to contain 1‐2 binding sites for the circPRMT5 region.^11^ Particularly, miR‐377 has been proved to be involved in the proliferation and metastasis of many types of tumours of digestive system.^12^, ^13^, ^14^, ^15^ Therefore, we suggested that circPRMT5 can bind to miR‐377 in the progression of CRC. Thus, the occupancy of Ago2 in the region of circPRMT5 was analysed by using RNA immunoprecipitation and showed that endogenous circPRMT5 pulled‐down from Ago2 cells was specifically abounded, indicating that circPRMT5 could incorporate into the RNA‐induced silencing complex in CRC (Figure 3C). In addition, we found that compared with the control miRNA, miR‐377 significantly reduced the luciferase reporter activities in CRC cells, while transfection of the miR‐377 showed little effect on luciferase activity, when the miRNA target sites were mutated (Figure 3D), suggesting that circPRMT5 may function as a sponge to miR‐377 in CRC. Moreover, when knockdown circPRMT5, the abundance of miR‐377 in circPRMT5 probe pull down fraction was very much decreased. In turn, when overexpressed circPRMT5, the enrichment of miR‐377 in circPRMT5 probe pull down fraction was significantly up‐regulated (Figure 3E). These data provided evidence that circPRMT5 is able to directly bind miR‐377. We further investigated whether circPRMT5 exerts its oncogenic function through sponge activity of miR‐377 by using miR‐377 inhibitor and revealed that miR‐377 inhibitor could functionally restore circPRMT5 silence‐suppressed CRC cells proliferation as determined by CCK‐8 analysis (Figure 3F) and colony formation assay (Figure 3G). What is more, the results of Western blotting and RT‐PCR also revealed that the oncogenic role of circPRMT5 in regulating cell cycle‐associated proteins *CyclinD*1 and CDK2 of CRC was dependent on the sponge activity of miR‐377 (Figure 3H,I). These data suggested that circPRMT5 can effectively suppress miR‐377 function to promote CRC progression.

### 3.5 CircPRMT5 regulates the miR‐377/E2F3 pathway in CRC cells

**Figure 4:**
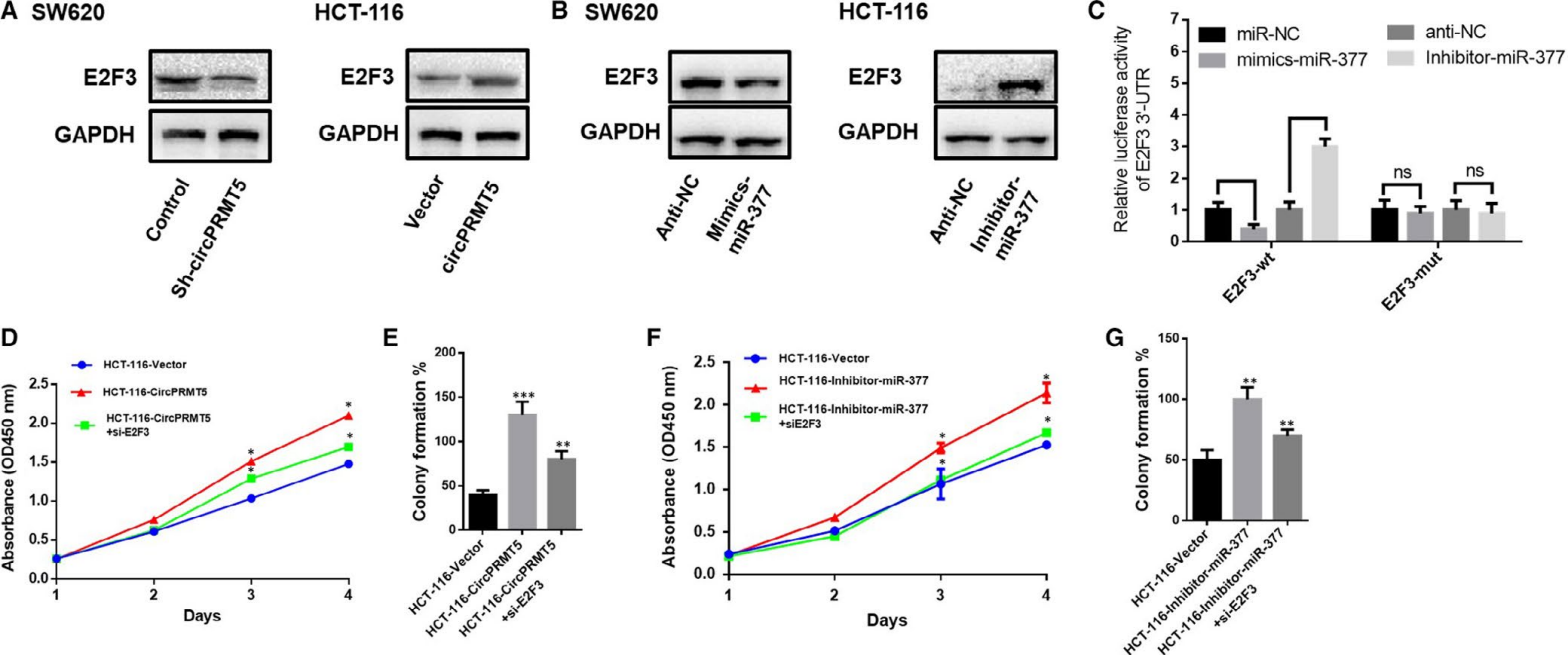
CircPRMT5 regulates the miR‐377/E2F3 pathway in CRC cells. (A). Protein expression of E2F3 of CRC cells overexpression of circPRMT5 or down‐regulation of circPRMT5 was determined by Western blotting; (B). protein expression of E2F3 of CRC cells transfected with miR‐377 mimics or inhibitor was determined by Western blotting; (C). Luciferase reporter assay for targeting the 3’‐UTR of E2F3 by miR‐377; (D). CCK‐8 assay of HCT‐116 cells transfected with control vector or circPRMT5 overexpression plasmid within or without cotransfected with E2F3 siRNA. Data are mean ± SD. *P < .05; (E). colony formation assay of HCT‐116 cells transfected with control vector or circPRMT5 overexpression plasmid within or without cotransfected with E2F3 siRNA. Data are mean ± SD. **P < .01, ***P < .001; **(**F). CCK‐8 assay of HCT‐116 cells transfected with miR‐377 inhibitor within or without cotransfected with E2F3 siRNA. Data are mean ± SD. *P < .05; (G). colony formation assay of HCT‐116 cells transfected with miR‐377 inhibitor within or without cotransfected with E2F3 siRNA. Data are mean ± SD. **P < .01, ***P < .001

It is reported that miR‐377 targets E2F3 in malignant tumours.^16^ To further found the exact mechanisms why circPRMT5 can suppress function of miR‐377 to promote CRC development, we have been suggested that circPRMT5 may be responsible for promoting the expression levels of miR‐377 downstream targets E2F3 by acting as a miR‐377 sponge. The results showed that decreased expression of circPRMT5 could significantly lead to decrease of E2F3 expression, while up‐regulation of circPRMT5 could promote E2F3 expression (Figure 4A). We then explored whether miR‐377 regulates the CRC cell proliferation by controlling the expression of E2F3, and found that miR‐377 mimic could reduce the protein levels of E2F3. Correspondingly, the E2F3 level was increased following the transfection with the miR‐377 inhibitor (Figure 4B). Meanwhile, targeted mutation of miR‐377 binding sites in the 3’UTR of E2F3 abrogated the miR‐377‐induced lessen in luciferase expression, showing that miR‐377 could directly targets E2F3 in CRC (Figure 4C). CCK‐8 and colony formation assay confirmed the effect of circPRMT5 up‐regulation can be reduced by E2F3 inhibition (Figure 4D,E), and the effect of miR‐377 inhibition can be suppressed by E2F3 inhibition (Figure 4F,G). Taken together, these data indicated that circPRMT5 may be involved in enhancing the expression levels of miR‐377 downstream targets E2F3 by functionally as a miR‐377 sponge.

## 4 DISCUSSION

This study demonstrated that circPRMT5 was an important up‐regulation circRNA in CRC, and higher expression of circPRMT5 showed poorer survival of CRC patients. CircPRTM5 exerts critical oncogenic role in CRC by sponging miR‐377 to induce E2F3 expression and promoting cell cycle‐associated proteins *cyclin*D1 and CDK2 expression.

Emerging evidence suggests that dysregulation of circRNAs plays a pivotal role in the development of malignancies.^17^, ^18^, ^19^, ^20^ Our results showed that up‐regulation circPRMT5 was a commonly oncogenic event in CRC, contributing to the cell proliferation by regulating cell cycle‐associated proteins *cyclin*D1 and CDK2. *Cyclin* D1 is an important cyclin to be elevated by growth factors during G1 phase of cell cycle and is considered to be a key mediator of extracellular signals that regulate cell growth.^21^, ^22^ Amplification of CDK2 also often occurred in tumorigenicity with high proliferation rate.^23^ Since *cyclin* D1 and CDK2 are the key cell cycle‐related genes controlling cell proliferation, our results confirmed that circPRMT5 could promote cell proliferation of CRC via regulating *cyclin* D1 and CDK2 expression.

It is well characterized that circRNAs function as miRNA sponges in the progression of tumorigenicity. To better understand the regulatory mechanism of circPRMT5 in CRC, we analysed the miRNAs known to be bound by circPRMT5, and found that miR‐377 could able to interact with circPRMT5 in CRC progression. MiR‐377 belongs to a large miRNA cluster whose expression is frequently silenced in human malignancies, such as neuroblastoma, ependymoma, gastro‐intestinal stromal tumours, osteosarcoma and prostate cancer.^24^, ^25^, ^26^, ^27^, ^28^ Further functional studies showed that miR‐377 inhibitor could functionally restore circPRMT5 silence‐suppressed CRC cells proliferation, and the oncogenic role of circPRMT5 in regulating cell cycle‐associated proteins *Cyclin*D1 and CDK2 of CRC was partly dependent on the sponge activity of miR‐377. These data confirmed that circPRMT5 can effectively extinguish function of miR‐377 to promote CRC progression. It is reported that miR‐377 can also directly target certain oncogenes to affect cell migration and invasion.^28^ Our results showed that silence of circPRMT5 could significantly reduce E2F3 expression, and the protein levels of E2F3 were declined when the miR‐377 mimic was added. E2F3 serves as a potential transcriptional inducer of cell cycle progression, and its amplification was significantly associated with tumour progression.^29^, ^30^ Our study showed that the proliferation effect of circPRMT5 up‐regulation can be reduced by E2F3 inhibition, and the effect of miR‐377 inhibition can be suppressed by E2F3 inhibition. These data indicated that circPRMT5 may be act as a miR‐377 sponge to enhance the expression of miR‐377 downstream targets E2F3. 

In conclusion, our study demonstrated that circPRTM5 was frequently up‐regulated in CRC tissues and patients with higher circPRTM5 levels showed a poorer overall survival. CircPRTM5 exerts critical regulatory role in CRC by sponging miR‐377 to induce E2F3 expression and promoting cell cycle‐associated proteins *cyclin*D1 and CDK2 expression, which opens up new insight into the potential treatment of CRC in humans.

## CONFLICT OF INTERESTS

The authors have no conflicts of interest to disclose.

## AUTHOR CONTRIBUTIONS

BRY, KD and CHY contributed equally. WNL designed the study. SJW, KD, YX and CHY performed the in vitro experiments. KD, LLX and CC performed the in vivo experiments. SJW and JHZ analysed the data and wrote the manuscript. KD and CC evaluated the histological features. WNL supervised the study.

## Data Availability

The data that support the findings of this study are available from the corresponding author upon reasonable request.
